# Psychological distress across the deployment cycle: comparing pre- and peri-pandemic trajectories

**DOI:** 10.1186/s12889-025-23746-5

**Published:** 2025-08-22

**Authors:** Antje H. Bühler, Gerd-Dieter Willmund

**Affiliations:** Bundeswehr Centre for Military Mental Health, Military Hospital Berlin, Scharnhorststr. 13, Berlin, 10115 Germany

**Keywords:** Pre- and peri-pandemic, Quarantine, Psychological distress, Military deployment

## Abstract

**Background:**

To prevent military personnel from becoming vectors of SARS-CoV-2 transmission during the pandemic, soldiers throughout the world were required to quarantine before and after deployment. This study examined whether deployment-related quarantining affected trajectories of psychological distress for German military personnel throughout the deployment cycle. Pandemic-specific mental health studies were criticised for lacking a pre-pandemic reference point. This study aims to address this gap, thus providing stronger evidence as to whether deployment-related quarantining affected psychological distress.

**Method:**

Pre-pandemic data were pooled with peri-pandemic data. The pre-pandemic sample consisted of 96 soldiers, and the sample from the pandemic group included 95 soldiers. Both groups completed the Brief Symptom Inventory (BSI)/Mini-Symptom Checklist (Mini-SCL) at three time points: two weeks before deployment, two weeks after deployment, and at a follow-up, three to six months post-deployment. The pandemic sample participated in both pre-deployment and post-deployment quarantines, completing the Mini-SCL five times. A two-way repeated-measures ANOVA assessed differences across deployment phases and time periods.

**Results:**

The repeated measures ANOVA revealed significant main effects for the within-subjects factor "deployment phases" and the between-subjects factor "pre- versus peri-pandemic period," with a significant interaction between both factors. Follow-up analyses showed 1) differing pre- and peri-pandemic trajectories of psychological distress across the deployment cycle and 2) higher levels of psychological distress during the peri-pandemic period, except for the two-week post-deployment measurement, which coincided with the end of post-deployment quarantine.

**Conclusions:**

While it is difficult to disentangle the specific impact of the pandemic versus deployment-related quarantine measures, the findings suggest that the pandemic itself had a more substantial impact on psychological distress than the quarantine measures. This study underscores the importance of selecting critical measurement time points, and utilising shorter, streamlined measures in future research.

## Introduction

The Covid-19 pandemic presented conflict-ridden countries and countries supplying stabilising forces to these countries with a dilemma: a lack of patrolling and rotation of accountable military forces would have left a power vacuum which could have been filled by irregular armed groups further threatening the vulnerable population in these fragile countries [1]. At the same time, troop rotations themselves posed a threat to the vulnerable population’s health becoming a potential contagion vector. Historic cases of pandemics or epidemics being attributed to troop rotations were the "Spanish" flu causing 20–50 millions of deaths in 1918–1920 after the first world war [2], and the introduction of vibrio cholerae by peacekeepers to Haiti in 2010 causing thousands of deaths [3, 4]. COVID-19 caused similar concerns. A lack of healthcare, health protection and delayed immunisation left the population in war-torn countries, mostly low-income countries, even more vulnerable to highly contagious and potentially life-threatening viruses such as SARS-Cov-2 [5].

In order to mitigate virus transmission into the critical areas and to maintain operational capabilities, on 4 April 2020 the United Nations Secretary-General suspended the rotation and deployment of all uniformed personnel (upon request of the member countries) until 30 June 2020 (7). The different bodies of the United Nations (UN), including the World Health Organization (WHO), UN Department of Peace Operations and Department of Operational Support, as well as Departments/Ministries of Defence worldwide issued force-specific health protection guidance for deployment and redeployment of individuals and units during the Covid-19 pandemic [6, 7]. One of the main non-pharmacological measures to contain infections for rotating troops worldwide was quarantining, a two-weeks long pre-deployment quarantine separating soldiers individually in a separate facility and a two-week home-based quarantine after returning home [6–8]. Depending on mission area and infectious dynamics, mandatory quarantining was adjusted over time until pre-deployment quarantining was officially suspended on 1 April 2022 by the United Nations Department of Peace Operations [8].

Although the terms "isolation" and "quarantine" are often used interchangeably by the general public to refer to strict confinement in a separate room and distancing from others, we apply them in their epidemiological sense."Isolation"involves separating individuals who have been confirmed to be infected with a virus, such as SARS-CoV-2, while "quarantine" applies to those who have been exposed to someone known to be infected with the virus. Additionally, pre-deployment and post-deployment quarantine measures have been enforced for military personnel, even in the absence of known exposure to an infected individual.

The enforced quarantine measures for rotating military personnel initially led to complaints from soldiers to the Parliamentary Commissioner of the German Armed Forces [9], who reported that the soldiers argued that the pre-deployment quarantine infringed upon their rights. As a result, we were particularly interested in examining how quarantine and the pandemic had affected psychological distress throughout the deployment cycle.

### Impact of quarantining on mental health

Several rapid reviews, systematic reviews, and two meta-analyses concluded that quarantining and isolation negatively affected mental health during epidemics or pandemics [10–16]. However, several limitations exist in the studies upon which these analyses were based: (a) a lack of differentiation between isolation and quarantining, (b) variability in the implementation of quarantining, and (c) methodological issues, including: (c1) the absence of pre-pandemic baseline measures, such as reference groups or reference trajectories for mental health, and (c2) reliance on post-hoc cross-sectional studies rather than prospective longitudinal studies. Research on the impact of home confinement and lockdowns further supports the latter concern: A meta-analysis of only longitudinal studies found considerable heterogeneity in results but concluded that most people exhibited psychological resilience to home confinement and lockdowns [17]. In contrast, systematic reviews that included cross-sectional studies and studies without control groups found significant negative mental health effects associated with home confinement and lockdowns [18–20].

Some of the methodological shortcomings discussed earlier are inherent to the very nature of pandemic quarantining. A more rigorous methodology, such as randomised controlled trials, could not be implemented for legal and ethical reasons. For example, suspending quarantine for a control group could have led to a worsening of the infection dynamics. Quarantining and isolation are often unplanned and occur unexpectedly, which complicates prospective measurements. Additionally, the unprecedented nature of the pandemic made it difficult to implement prospective research designs starting from a pre-pandemic baseline.

There is a notable lack of research on deployment-related quarantines for soldiers with few exceptions [21–24], which differ in key ways from civilian quarantines. In military contexts, quarantines during the COVID-19 pandemic and the Ebola epidemic in West Africa were primarily imposed due to the deployment and/or redeployment of personnel, rather than as a result of confirmed infections (e.g., SARS-CoV-2). Deployment-related quarantines, therefore, present a unique opportunity to examine the specific impact of quarantine itself. Due to integrated deployment planning, prospective longitudinal designs can be implemented, allowing for the disentangling of quarantine-specific stressors from the potential traumatic stressor of a life-threatening infection in the case of isolation. An additional advantage of studying deployment-related quarantines during the COVID-19 pandemic, both in terms of soldiers' wellbeing and research methodology, is that many of the quarantine-related stressors were mitigated by the quarantine protocols of the Bundeswehr, which addressed issues such as inadequate information, insufficient supplies, financial hardships, and stigma in a standardised manner. German military personnel were regularly updated on the SARS-CoV-2 virus and the pandemic. At quarantine facilities, military logistics teams briefed the deploying soldiers on the quarantine protocol during the in-processing at the respective hotel facilities. Hotel staff and military logistics teams ensured the provision of daily necessary supplies. Medical care and a psychological hotline were also made available. Regular salaries were maintained, and stigmatisation was less likely, as deployment-related quarantining was not linked to individual behaviour or infections, but rather to mandatory deployment policies applied to all deploying soldiers.

In our previous work on deployment-related quarantines, our main concern was to provide real-time advice to military leadership and military psychologists on which quarantine-related factors to address if quarantine adherence declined or psychological distress increased in the short-term. We focused specifically on changes in psychological distress and quarantine adherence from the start of pre-deployment quarantine [23, 24] and examined the role of various quarantine-related risk and resilience factors. These included feeling adequately informed about COVID-19, perceived infection risk, perceived benefits of quarantine, clear communication of quarantine protocols, fulfilment of intimacy and bonding needs, perceptions of social norms, perceived stigma, practicality of quarantine conditions (e.g., access to daily essentials and medical care), financial challenges, boredom, and the role of health-promoting leadership. Our findings emphasized that clear communication of quarantine protocols was a key protective factor, followed by perceived social support and perceived unit cohesion during pre-deployment quarantine, which were associated with lower levels of both short-term psychological distress and long-term psychological distress post-deployment [23, 24].

Contrary to reviews and meta-analyses suggesting a negative impact of quarantine on civilian mental health [14–16], we observed no significant increase in peri-pandemic psychological distress during the deployment cycle for soldiers undergoing pre-deployment quarantine. However, we did not analyse the trajectory of distress for a smaller subsample of soldiers who underwent both pre- and post-deployment quarantine. Additionally, one limitation of our previous work on the impact of military deployment-related quarantining is the lack of a control group, making it difficult to account for the impact of deployment phases, the pandemic, and quarantining. As a result, a robust assessment of quarantine's impact is lacking. It remains unclear whether post-deployment distress might have decreased further without quarantine or how both pre- and post-deployment quarantines affect long-term distress.

This study aims to address these methodological gaps by introducing a pre-pandemic control group, which provides a reference trajectory of psychological distress across the deployment cycle for comparison with the peri-pandemic trajectory. By controlling for the effects of deployment phases and the pandemic period, this approach allows for more robust, evidence-based conclusions regarding the psychological impact of deployment-related quarantining.

### Mental health across the deployment cycle

Deployment itself has been considered a risk factor for trauma-related mental health disorders, while prevalence and incidence rates vary largely, depending on methodology, operational theatre and target group [25, 26]. However, there seems to be a growing body of evidence that it is not deployment per-se that has a negative impact on mental health, it is rather the combat experience [25, 26]. This finding also applies to German troops, as prevalence rates of mental health disorders do not differ significantly between pre- and post-deployment [27, 28]. While overall prevalence rates are lower for military than the civilian population (14% versus 20%), incidence rates of anxiety disorders and PTSD are higher for soldiers with combat experience [27, 28].

Furthermore, the role of sociodemographic factors, including age, sex/gender, rank, partnership and deployment-related factors such as previous deployment experience and length of deployment were analysed. While there is convergent evidence suggesting that deployment length is a risk factor for mental health [29], the evidence regarding previous deployment experience [30–33] and sociodemographic factors is mixed. Specifically, it remains unclear whether younger age (< 30), older age (> 50), female gender [34–36, 36], lower rank, and being single are consistent risk factors for mental health [36].

While different trajectories of mental health across the deployment cycle for a US-American military sample have been identified [37], data on the typical pre-pandemic trajectories of psychological distress, mental health and resilience across the deployment cycle for a German military sample is missing so far.

To summarize, we are interested in answering the following questions:

1. Do levels of psychological distress differ between soldiers deployed before the pandemic and those deployed during the pandemic?
2. Do the patterns of psychological distress across the deployment cycle differ between the pre-pandemic and peri-pandemic groups?
3. If the overall pattern is similar, do the size and direction of changes in psychological distress between deployment phases differ between the two groups?
4. Does participation in deployment-related quarantine (in the peri-pandemic group) appear to contribute to increased psychological distress?

## Methodology

### Research design

We compared the trajectory of psychological distress across the deployment phases of the pandemic period from 2021–2022 with the corresponding pre-pandemic psychological distress trajectory from 2013–2016. The criterion for selecting this time period was the alignment of the three measurement time points across the deployment cycle for German troops with the research instrument, the Brief Symptom Inventory. The only available pre-pandemic data fulfilling this criterion was collected in this time period (2013–2016). Points of measurement were taken two weeks prior to deployment, two weeks after returning home, and at a follow-up three to six months post-deployment. For the pandemic sample, these measurement points coincided with the quarantine period, with t1 representing the in-processing into pre-deployment quarantine and t4 marking the end of post-deployment quarantine. In line with the measurement points of the pre-pandemic sample, which had been taken two weeks before and two weeks after return, we compared t1 and t4 for the main analysis (see Table 1). In this analysis, we did not examine short-term differences in psychological distress between the beginning and end of deployment-related quarantines, as we had previously published the results of non-significant differences in psychological distress between the start and end of the respective quarantines (t1 versus t2 and t4 versus t3)" [23, 24].

**Table 1:** Pre-pandemic and peri-pandemic assessments across the deployment cycle

	**Pre-deployment**		**Post-deployment**		
	T1	T2	T3	T4	T5
2013–16	X			X	X
2021/22	X quarantine	X quarantine	X quarantine	X quarantine	X

T1: two weeks before deployment, peri-pandemic: beginning of pre-deployment quarantine

T2: peri-pandemic: at the end of pre-deployment quarantine, 1–2 days before being deployed

T3: peri-pandemic: immediately when entering post-deployment quarantine

T4: two weeks after returning home, peri-pandemic: end of post-deployment quarantine

T5: Pre-pandemic: six months after redeployment, peri-pandemic: two to three months post-deployment

### Survey: measures

The choice of instruments for our surveys in the pre- and peri-pandemic research projects was guided by varying research objectives, recruitment options, and the availability of recommended measures, which differed between the two projects at the time. The pre-pandemic research initiative studied the impact of military deployment and combat exposure on mental health, comparing deployed to non-deployed soldiers’ mental health trajectories across various mental health measures, including PTSD, depression, anxiety, substance abuse, and changes in brain function. The BSI served as a change sensitive measure to track potential changes in psychological distress, capturing sub-clinical and clinically relevant changes in symptoms and their severity regardless of the specific mental disorder. The peri-pandemic study prioritised providing timely advice to military leadership on addressing factors related to increased psychological distress or decreased quarantine adherence, focusing on factors like social support, unit cohesion, and quarantine-related risk and resilience factors, rather than diagnosing specific mental health disorders. We considered the Mini-SCL (BSI-18) the most effective and efficient measure to indicate the necessity of timely leadership consultations. For the purpose of this analysis, we describe only the research instruments and measures used for comparing the level of psychological distress and the trajectories of psychological distress across the deployment phases between the soldiers deployed before versus during the pandemic.

#### Psychological distress

##### Brief Symptom Inventory (BSI)/Mini-symptom checklist

The 53-item BSI, previously chosen for the pre-pandemic sample, was matched by the shorter Mini-SCL, the German version of the 18-item BSI-18, for the peri-pandemic sample. The reason for the choice was that they offer a change-sensitive tool for assessing psychological distress across the full spectrum, from healthy individuals to those with subclinical or diagnosable mental health disorders, without focusing on specific symptoms or diagnoses [38]. By measuring distress over the past seven days, it also captures a relevant period for assessing the short-term impact of quarantine. The Mini-SCL includes three subscales: depression, anxiety, and somatization [38–41]. The Global Severity Index (GSI) measures overall psychological distress taking into account the number and intensity of all symptoms for the BSI and the Mini-SCL. Despite controversies regarding the factorial and discriminant validity of these scales, the Global Severity Index (GSI) of both tools demonstrates high internal consistency (Cronbach’s α > 0.9), good test–retest reliability (0.68 ≤ *r* ≤ 0.91), and strong convergent validity with symptom severity across mental health disorders [42, 43]. The GSI also strongly correlates with its subscales: depression (*r* = 0.87), anxiety (*r* = 0.88), and somatization (*r* = 0.79) [44]. Cross-cultural studies recommend using the GSI as the primary, reliable, and valid indicator of psychological distress, rather than the subscales [43]. For comparing pre- and peri-pandemic data, high correlations between the GSI scores of the BSI-18 (Mini-SCL) and BSI (*r* = 0.91 to 0.96) [39], along with available German sex-specific population norms (T-values), suggest that the index is equivalent allowing for distress levels of both cohorts to be compared. Furthermore, the Mini-SCL takes 1–2 min to complete, thereby reducing the risk for dropout and missing values for the peri-pandemic sample with five points of measurement.

#### Critical traumatic incidents during deployment

##### Combat Exposure Scale (CES)/Life Events Checklist (LEC-5)

were assessed by the 33-item German adapted version of the Combat Exposure Scale (CES) of the Military Health Advisory Team (MHAT) in the pre-pandemic research project and the 16-item Life Events Checklist (LEC-5) of the Posttraumatic Stress Disorder Checklist for DSM-5 (PCL-5) for the peri-pandemic research project. The reasons for choosing different instruments were (a) their availability when designing the study, and (b) the research purpose, which involved identifying either pre-pandemic combat-related stressors or a more comprehensive range of stressors during the pandemic, including illness due to infections, with the constraint of a shorter completion time applying to the peri-pandemic sample. The LEC-5 assesses closeness to the event and whether it occurred during one’s professional duty (e.g., deployment), rather than the frequency of the event, as in the CES. We harmonised the CES and the LEC-5 by simplifying the scale into a dichotomous format: 'deployment-related traumatic events: yes – no’.

#### Sociodemographic and deployment-related factors

The following additional data was obtained in both samples: age, sex/gender, partnership, children, rank, deployment experience (number of previous deployments and accumulated days of previous deployments), area and mission of deployment and deployment length.

### Plan for analysis

All analyses were conducted using SPSS 29. The required sample size was calculated with the assistance of GPower [45]. When unavailable in SPSS 29, effect sizes and confidence intervals (CIs) were computed manually using the website https://effect-size-calculator.herokuapp.com/ for partial eta squared and omega squared.

*Analysis Procedure*: We planned to test four statistical hypotheses:

1. Soldiers’ levels of psychological distress during the deployment cycle differ significantly between the pre- and peri-pandemic deployment cycles.
2. Soldiers’ levels of psychological distress differ significantly between different deployment phases.
3. Pre- and peri-pandemic trajectories of soldiers'psychological distress across the deployment cycle differ significantly: There is a significant interaction between the predictors ‘pre- versus peri-pandemic period’ and the ‘deployment phases’.
4. There is a greater increase or smaller decrease psychological distress between the deployment phases for the pandemic than the pre-pandemic group: a) There is a greater increase or smaller decrease in peri-pandemic psychological distress between pre- and post-deployment measurements, two weeks before deployment and two weeks post-deployment (t1 versus t4). b) Post-deployment: There is a greater increase or a smaller decrease in peri-pandemic distress three to six months post-deployment as compared to two weeks post-deployment (t4 versus t5).

To account for variation across time and groups, a two-way repeated measures ANOVA was conducted, with psychological distress (measured by BSI/Mini-SCL) as the dependent variable. The analysis included the pre-pandemic versus peri-pandemic period as the between-subjects factor, deployment phases as the within-subjects factor, and their interaction. If significant differences were found between the pre- and peri-pandemic samples with respect to any relevant sociodemographic or deployment-related variables, these were included as covariates in the analysis. The variables tested for significant differences included age*,* sex/gender*,* marital status *(being single),* rank*,* deployment experience (number of previous deployments, accumulated deployment experience in days), length of the current deployment in months), as well as exposure to deployment-related traumatic events, such as life-threatening combat.

Should the pre- and peri-pandemic samples differ significantly across a number of inter-correlated sociodemographic and deployment-related variables, concerns regarding multicollinearity and model overfitting may arise, given the small sample size. These issues could undermine the stability and accuracy of the subsequent analysis. To preserve the majority of the variance while mitigating the complexity and multicollinearity, a principal component analysis (PCA) should be performed in this case. By incorporating the retained principal component scores as covariates in the MANOVA, the essential information from the original variables was preserved, while facilitating a more robust and interpretable analysis.

Interpretation: Significant within-subject effects were followed up by Helmert contrasts. However, in the event of a significant interaction between the within-subjects factor *deployment phases* and the between-subjects factor *pre- versus peri-pandemic period*, any significant main effects for *deployment phases* and/or *pre- versus peri-pandemic period* were not interpreted without first analysing the interaction between these two factors. If a significant interaction was found, the trajectories of psychological distress were analysed separately for the pre- and peri-pandemic periods. Similarly, differences in psychological distress levels between the pre- and peri-pandemic periods were analysed at each measurement point separately before drawing conclusions about overall differences between the periods.

Interpretation of significant within-subjects and between-subject effects was conducted with the help of estimated means and profile plots.

Given that the hypotheses underlying the research questions were non-directional (i.e., difference versus no difference), the Type 1 error rate was set at α = 0.05. Pairwise comparisons were Bonferroni-corrected by SPSS.

### Required sample size

Due to non-directional testing, the α-error was set at α = 0.05 for the two-way repeated measures ANOVA. A minimum sample size of 74 participants was needed to provide sufficient power for detecting differences of an effect size f = 0.15 and a sample of 164 for a smaller effect size of *f* = 0.1 (F-tests**—**ANOVA: Repeated measures, within-between interaction, α err prob = 0.05, power (1-β err prob) = 0.80, number of groups = 2, number of measurements = 3, Corr among rep measures = 0.5).

### Recruitment

Participation was voluntary with no incentives. Participants were informed in writing and given contact details for further questions. Consent, including follow-up assessments, was obtained in writing, and participants could withdraw at any time. Anonymity and data matching were ensured via pseudonymisation.

The pre-pandemic sample was recruited between June 2013 and August 2016. Soldiers were informed about the study through project presentations during pre-deployment training and by military medical staff during pre-deployment assessments. Interested participants met with a study team member for detailed information before enrolment.

The peri-pandemic sample was recruited from February 2021 to March 2022 during in-processing into pre-deployment quarantine. Consent was adapted to the quarantine setting, with verbal and written information provided during in-processing. Participants were given study packages to ensure anonymity, with phone numbers for further consultation.

While neither group received reimbursement, the conditions under which participation took place differed considerably: In the pre-pandemic study, soldiers participated during official duty hours, completing surveys, interviews, and brain scans, with personal contact to a study team member for additional support. In contrast, peri-pandemic participation was likely driven by boredom during pre-deployment quarantine, with the added challenge of maintaining engagement post-deployment through long-distance surveys. Personal face-to-face contact with a study team member was not available in the peri-pandemic sample. To minimise dropouts and missing data, the post-deployment follow-up survey for the pandemic sample excluded scales related to quarantine adherence and quarantine-associated factors, thereby keeping the completion time within 10 min.

### Attrition and missing data: accounting for potential bias

High attrition is common in military studies, due to a lack of incentives and logistical barriers such as upcoming deployments, training, or relocations. Younger soldiers and those of lower rank tend to drop out more frequently. Although Little’s MCAR tests indicated that the data were missing completely at random in both samples (pre-pandemic: Chi-Square = 30.09, *df* = 46, *p* = 0.966; peri-pandemic: Chi-Square = 3.43, *df* = 4, *p* = 0.489), imputations were not conducted due to the high attrition rate. Furthermore, we tested for relationships between drop-out and sociodemographic and deployment-related variables.

For the pre-pandemic sample, 158 participants were initially recruited; 62 (39,24%) dropped out, resulting in 96 participants at six months post-deployment. Sociodemographic and deployment-related variables were not significantly associated with attrition, although a non-significant trend suggested younger soldiers might have slightly higher rates (*r* = −0.133, *p* = 0.051).

For the peri-pandemic sample, 129 participants were recruited, with 34 (26.36%) dropping out by three months post-deployment. Dropout was not related to age (r = −0.092, *p* = 0.298), sex/gender (*r* = −0.064, *p* = 0.472), or rank (*r* = −0.015, *p* = 0.868). More deployment experience was slightly positively associated with study completion (number of deployments: *r* = −0.194, *p* = 0.029; accumulated deployment experience: *r* = −0.183, *p* = 0.042).

## Results

### Sample: participant characteristics

No significant differences were found in respect to the distribution of gender across the pre-pandemic and the pandemic sample (*X*^2^ [1, *N* = 192] = 1.85, *p* = 0.174), the percentage in a partnership (*X*^2^ [1, *N* = 191] = 1.06, *p* = 0.303) or the percentage with children (*X*^2^ [4, *N* = 189] = 8.24, *p* = 0.083. However, the pandemic sample was older (*F*[1,185] = 16.41, *p* < 0.001), was marked by a larger proportion of higher ranks (*X*^2^[2, *N* = 189] = 7.04, *p* = 0.030), had deployed more often (*F*[1,185] = 11.95, *p* < 0.001) and had accumulated more deployment experience in days (*F*[1,185] = 4.12, *p* = 0.044 (see Table 2 for more details), although they deployed for a shorter period during the pandemic (*F*[1,185] = 168.35, *p* < 0.001).

**Table 2:** Comparison of sociodemographic and deployment-related characteristics

	***N***	**Pre-pandemic sample** *n* = 96	***n***	**Pandemic sample** *n* = 95	***n***
Age***	187	19-59 years	95	20–63 years	92
		*M*=33.41		*M* = 38.52	
		*SD*=8.73		*SD* = 9.13	
		*Mdn* = 30		*Mdn* = 38	
Sex/Gender	191		96		95
Ratio male:female		91.7%:8.3%	88:8	85.3%:14.7%	81:14
Rank*	189		96		93
Enlisted		14.6%	14	5.2%	5
NCO		54.2%	52	46.9%	45
Officers		31.3%	30	44.8%%	43
In a Partnership	191		96		95
Yes		76%	73	81.3%	78
Children (total)	189		96		93
No children		61.5%	59	41.7%	40
One child		16.7%	16	18.8%	18
Two children		17.7%	17	26.0%	25
Three or more		4.1%	4	10.4%	10
Previous deployments***	187		95		92
None		41.7%	40	22.9%	22
One		24.0%	23	20.8%	20
Two		12.5%	12	11.5%	11
Three		6.3%	6	11.5%	11
Four		7.3%	7	7.3%	7
Five or more		8.2%	8	24.7%	24
Days deployed*	187		95		92
		≤ 1400 days		≤ 1800 days	
		*M* = 196.63		*M* = 278.23	
		*SD* = 265.34		*SD* = 345.21	
		*Mdn* = 119.50		*Mdn* = 150	
Planned length of deployment***	187	3–9 months	95	1–8 months	92
		*M* = 5.22		*M* = 2.75	
		*SD* = 1.05		*SD* = 1.50	
		*Mdn* = 5		*Mdn* = 2	

^*^p <.05, **p <.01, ***p <.001

**Fig. 1:**
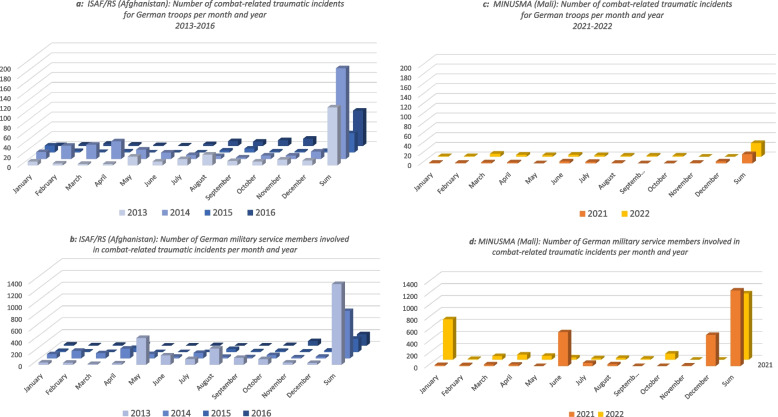
**a**-**d** Combat-related traumatic events and involved German military service members during deployment per month: ISAF/RS (Afghanistan) from 2013–2016 versus MINUSMA (Mali) from 2021–2022 Legend: ISAF = International Security Assistance Mission, RS = Resolute Support, MINUSMA = Multidimensional Integrated Stabilisation Mission. Combat-related incidents are presented on a scale from 0–40 incidents/months (**a** and **c**) and German soldiers involved in the incidents are presented on a scale from 0–800 soldiers involved in the corresponding incidents/months (**b** and **d**). The number of traumatic incidents or involved military service members are summed up per year (sum)

The deployment missions and areas differed slightly between the pre- and peri-pandemic samples, with no clear indication of which sample was more exposed to traumatic critical incidents on an individual basis. The pre-pandemic sample had a larger number of soldiers deployed to Afghanistan, while the peri-pandemic sample saw more personnel deployed to Mali and Iraq (see Table 3). Therefore, we also present the frequency of combat incidents per month and the number of German military personnel involved in these incidents per month for the most prevalent missions during the relevant pre-pandemic years (2013–2016) and peri-pandemic years (2021–2022). The missions presented include the International Security Assistance Force/Resolute Support in Afghanistan (ISAF/RS, 2013–2016) and the United Nations Multidimensional Integrated Stabilization Mission in Mali (MINUSMA, 2021–2022) (see Fig. 1a-d).

**Table 3:** Missions and mission areas the study participants deployed to before and during the COVID-19 pandemic

	Pre-pandemic (*n* = 96) (2013–2016)		Peri-pandemic (*n* = 95) (2021–2022)	
**Country: Mandate**	Frequency	Percent	Frequency	Percent
Afghanistan: ISAF/RS/UNAMA^1^	54	56.3	24	25.3
Kosovo: KFOR^2^	17	17.7	2	2.1
Turkey: Active Fence	1	1	0	0
Mali: MINUSMA/EUTM^3^	9	9.4	32	33.7
Niger: MINUSMA GAZELLE	0	0	7	7.3
Irak: CD/CBI^4^	1	1	11	11.6
Libanon: UNIFIL^5^	0	0	2	2.1
Estonia: VAPB^6^	0	0	15	15.8
Lithuania: efP. BTG^7^	0	0	1	1.1
Missing	14	14.6	1	1.1
Total	96	100.0	95	100.0

^1^*ISAF* International Security Assistance Force, RS = Resolute Support,

^2^*KFOR* Kosovo Force

^3^*MINUSMA* United Nations Multidimensional Integrated Stabilization Mission in Mali, EUTM = European Union Training Mission Mali

^4^*CD/CBI* Counter Daesh/Capacity Building

^5^*UNIFIL* United Nations Interim Force in Lebanon

^6^*VAPB* Strengthening Air Policing Baltic states (Verstärkung Air Policing Baltikum)

^7^*eFP* enhanced Forward Presence (Lithuania), BTG: Battle Group

#### Traumatic incidents during deployment

To identify incidents during the previous deployment, we compared incidents reported at t1 and t4. However, depending on the specific item or incident, up to 18% fewer incidents were identified post-deployment compared to pre-deployment, raising concerns about the reliability (and consequently the validity) of the data, which has been supported by a recent analysis of the test–retest stability of the LEC-5 [46]. This analysis concluded that the LEC-5 did not allow to distinguish between trauma-exposed and non-exposed individuals [46], thereby contradicting a previous positive assessment of the LEC-4 [47].

Given these findings, which call into question both the reliability and the validity, we therefore excluded'traumatic incidents during deployment/combat experience'as a covariate in the two-way ANOVA with repeated measures. Instead, we provide a purely descriptive comparison of previously reported traumatic incidents as documented two weeks post-deployment (see Table 4). We advise caution in treating the reported events as an objective record and, even more so, in attributing them solely to the previous deployment.

**Table 4:** Comparison of critical incidents/traumatic events between pre- and peri-pandemic sample

**Traumatic event**	**Assessment**			
	**Pandemic**	**Pre-pandemic**		
	**Life Events Checklist** **(LEC-5) of the PCL**^**1) 2)**^	**Combat Experience Scale (CES) of the Deployment Risk and Resilience Inventory (DRRI)**^**2)**^		
Experience of having been attacked	attacked by weapon	attacked	attacked by small arms	attacked by artillery, rockets or mortars
	12.5%	24%	21.6%	27.8%
Combat	Combat activities (10)	Shot at opponent (16)	Close combat (18)	Ordered gunfire/shelling
	25%	14.4%	1%	3.1%
Life-threatening illness or injury	Life-threatening illness or injury	Knew someone who was severely injured or killed	Saw dead or wounded soldiers	
	8.3%	21.6%	23.6%	
Violent death	Sudden violent death	Knew someone who was severely injured or killed	Saw dead or wounded soldiers	
	10.4%	21.6%	23.6%	
Severe human suffering	Severe human suffering	Saw wounded women or children and could not help		
	13.5%	37.5%		
Caused death or severe injury	Caused death or severe injury	Responsible for death of opponent		
	7.3%	1%		
Traumatic incident during deployment	36%	60%		

^1^Caution should be exercised due to limited reliability of the measurements

^2^For the LEC-5 only duty-related incidents were counted

^3^Events were dichotomised (yes = 2, no = 1)

### Preparing covariates for factorial ANOVA with repeated measures: PCA

#### Sociographic and deployment-related characteristics: regression factors based on PCA

The sociodemographic and deployment-related variables that differed significantly between the pre- and peri-pandemic samples were inter-correlated, necessitating the consideration of multicollinearity. The number of previous deployments and accumulated days deployed were highly positively correlated (*r* = 0.956, *p* < 0.001, *N* = 190): Negative correlations were observed between planned length of deployment and deployment experience (times deployed: *r* = −0.276, *p* < 0.001, *N* = 190; accumulated days deployed *r* = −0.149, *p* = 0.040; *N* = 189). Older age was positively associated with rank (*r* = 0.363, *p* < 0.001, *N* = 188) and deployment experience (times deployed; *r* = 0.603, *r* = 0.553, *N* = 190, *p*s < 0.001), but negatively related to planned length of mission (*r* = −0.236, *p* = 0.001, *N* = 190).

In accordance with the analysis plan, a principal component analysis (PCA) with varimax rotation was conducted, including the variables age, rank, number of previous deployments, accumulated deployment experience in days, and planned length of deployment. This was done to reduce the number of factors and account for the correlations between the covariates. Based on the eigenvalues and scree plot, a two-factor solution was identified, explaining 68.99% of the variance (see Table 5 for the rotated factor solution).

**Table 5:** Principal component analysis^a^ for covariates

**Rotated component matrix**		
	Components	
	1	2
Lifetime deployment (accumulated days)	.946	
Number of previous deployments	.912	.164
Age	.580	.513
Rank		.870
Length of mission	-.173	-.554

Extraction Method: Principal Component Analysis

Rotation Method: Varimax with Kaiser Normalization

^a^Rotation converged in 3 iterations

The saved scores of the two regression factors were then entered as covariates into the factorial ANOVA with repeated measures, in order to control for differences between the samples.

### Main analysis: factorial ANOVA with repeated measures

As per the planned analysis, a factorial repeated measures ANOVA was conducted with psychological distress as the dependent variable. The within-subjects factor was deployment phases, and the between-subjects factor was pre- versus peri-pandemic period**.** Two regression factors, derived from a principal component analysis (PCA) with the variables age**,** rank**,** length of deployment**,** number of previous deployments, and accumulated deployment experience (measured in days), were included as covariates.

Assumptions were checked prior to analysis. The assumption of sphericity was not violated, as confirmed by Mauchly's test (χ^2^(2) = 0.36, *p* = 0.835), nor were assumptions of equal error variances violated for the three measurement points, as indicated by Levene's test (0.02 ≤ F(1, 182) ≤ 2.98, 0.086 ≤ *p*s ≤ 0.894).

The main effect for the within-subjects factor deployment phases was highly significant, *F*(2, 360) = 10.54, *p* < 0.001, η^2^ = 0.06, 90% CI [LL = 0.02, UL = 0.09], ω^2^ = 0.05, as was the main effect for the between-subjects factor pre- versus peri-pandemic period, *F*(1, 180) = 13.65, *p* < 0.001, η^2^ = 0.07, 90% CI [LL = 0.02, UL = 0.13], ω^2^ = 0.06. However, the interaction between deployment phases and pre- versus peri-pandemic period was also significant, *F*(2, 360) = 4.86, *p* = 0.008, η^2^ = 0.03, 90% CI [LL = 0, UL = 0.06], ω^2^ = 0.02, which necessitated caution in interpreting both significant main effects without considering the significant interaction. Instead, the trajectories of psychological distress had to be analysed separately for the pre- and the per-pandemic sample, before interpreting the main effect as a common trajectory of psychological distress across the deployment cycle. In the same line, differences in the pre- and peri-pandemic level of psychological distress had to be analysed for each point of measurement separately before drawing the conclusion that the peri-pandemic level of psychological distress across the deployment cycle is higher than the pre-pandemic level.

The interpretation of the interaction was facilitated by the absence of significant main effects and interactions for the covariates. Specifically, no significant effect was found for regression factor 1 (*F*(1, 180) = 1.63, *p* = 0.203, η^2^ = 0.01, 90% CI [LL = 0, UL = 0.04], ω^2^ = 0), nor for regression factor 2 (*F*(1, 180) = 0.59, *p* = 0.445, η^2^ = 0.00, 90% CI [LL = 0, UL = 0.03], ω^2^ = 0). Additionally, no significant interaction effects were observed for regression factor 1 (*F*(2, 360) = 1.19, *p* = 0.307, η^2^ = 0.01, 90% CI [LL = 0, UL = 0.02], ω^2^ = 0), or regression factor 2 (*F*(2, 360) = 0.11, *p* = 0.896, η^2^ = 0.00, 90% CI [LL = 0, UL = 0], ω^2^ = 0).

Within-subjects contrasts revealed a significant interaction for deployment phases ‘two weeks pre-deployment’ and ‘two weeks post-deployment’ and ‘pre- versus peri-pandemic period’, indicating that changes in psychological distress between the deployment phases ‘two weeks pre-deployment’ and ‘two weeks post-deployment’ depended on the pre- or peri-pandemic period, *F*(1,180) = 9.79, *p* = 0.002, η^2^ = 0.05, 90% CI [LL = 0.01, UL = 0.11], ω^2^ = 0.05. No significant interaction was found for the deployment phases ‘two weeks post-deployment’ and ‘three to six months post-deployment’ and the ‘pre- versus peri-pandemic period’, *F*(1,180) = 2.48, *p* = 0.119, η^2^ = 0.01, 90% CI [LL = 0, UL = 0.05], ω^2^ = 0.01. Therefore the significant within-subjects contrast for the two post-deployment levels of the within-subjects factor deployment phases could be interpreted (*F*(1, 180) = 5.88, *p* = 0.016, η^2^ = 0.03, 90% CI [LL = 0, UL = 0.08], ω^2^ = 0.03): Psychological distress slightly decreased from two weeks post-deployment (*M* = 44.28, *SE* = 0.75) to three to six months post-deployment (*M* = 42.79, *SE* = 0.77).

**Fig. 2:**
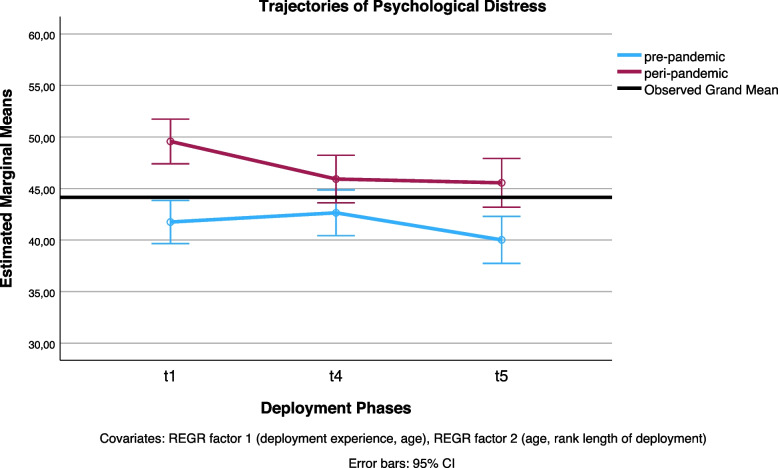
Psychological distress across the deployment cycle: pre-pandemic versus pandemic period Legend: t1: two weeks before deployment, peri-pandemic: beginning of pre-deployment quarantine. t4: two weeks after returning home, peri-pandemic: end of post-deployment quarantine. T5: Pre-pandemic: six months after redeployment, peri-pandemic: two to three months post-deployment

To aid in the interpretation of the significant interaction of the within-subjects contrast, we refer to the interaction plots (see Fig. 2) and the results of Bonferroni-corrected pairwise comparisons (see Table 6 for pre- and peri-pandemic estimates of each point of measurement).

**Table 6:** Pre- and peri-pandemic psychological distress across the deployment cycle

Measure: Sex-sensitive T-values for Brief Symptom Inventory (BSI)/Mini-Symptom Checklist (Mini-SCL)					
Pre-peri-pandemic	Deployment Phases	*M*	*SE*	95% CI	
				LL	UL
Pre-pandemic	T1	41.75^a^	1.06	39.66	43.84
	T4	42.64^a^	1.12	40.43	44.86
	T5	40.02^a^	1.15	37.74	42.30
Peri-pandemic	T1	49.57^a^	1.10	47.40	51.74
	T4	45.92^a^	1.17	43.62	48.22
	T5	45.55^a^	1.20	43.19	47.92

^a^Covariates appearing in the model: REGR factor 1, REGR factor 2

T1: two weeks before deployment, peri-pandemic: beginning of pre-deployment quarantine

T4: two weeks after returning home, peri-pandemic: end of post-deployment quarantine

T5: Pre-pandemic: six months after redeployment, peri-pandemic: two to three months post-deployment

As for the interpretation of the significant interaction found by the first within-subjects contrast: In the peri-pandemic sample, the level of psychological distress was lower two weeks post-deployment, coinciding with the end of post-deployment quarantine, than two weeks pre-deployment (*M*_t1-t4_ = −3.65, *SE* = 0.98, *p* < 0.001). For the pre-pandemic sample, in comparison, the very small increase in psychological distress between the two points of measurement was not significant (*M*_t1-t4_ = 0.89, *SE* = 0.94, *p* = 1).

To follow up on the interaction between deployment phases and pre- versus peri-pandemic period, we also explored whether the main effect of the pandemic could be observed across all three deployment phases. Specifically, psychological distress was significantly higher for the pandemic sample compared to the pre-pandemic sample during both the pre-deployment phase (*M*_t1_ = 7.82, *SE* = 1.64, *p* < 0.001, *F*(1,180) = 22.81, *p* < 0.001, η^2^ = 0.11, 90% CI [LL = 0.05, UL = 0.19], ω^2^ = 0.11) and six months post-deployment (*M*_t5_ = 5.54, *SE* = 1.78, *p* = 0.002, *F*(1,180) = 9.63, *p* = 0.002, η^2^ = 0.05, 90% CI [LL = 0.01, UL = 0.11], ω^2^ = 0.05). However, two weeks post-deployment, coinciding with the end of post-deployment quarantine for the pandemic sample, the same trend remained non-significant (*M*_t4_ = 3.28, *SE* = 1.74, *p* = 0.061 *F*(1,180) = 3.56, *p* = 0.061, η^2^ = 0.02, 90% CI [LL = 0, UL = 0.06], ω^2^ = 0.01).

## Discussion

We were interested in whether the level of psychological distress differed before and during the COVID-19 pandemic for deployed soldiers, whether there was a common trajectory of psychological distress with similar patterns of increases or decreases across the deployment phases, or whether the peri-pandemic trajectory of psychological distress during the deployment cycle differed from the corresponding pre-pandemic trajectory, and whether deployment-related quarantining increased medium- and long-term psychological distress.

Overall, pre-pandemic levels of psychological distress in deployed soldiers were lower than peri-pandemic levels, with the exception of a non-significant trend observed two weeks post-deployment, which coincided with the conclusion of post-deployment quarantine for the pandemic sample. Before the pandemic, soldiers displayed less severe symptoms than the pre-pandemic average population. However, during the pandemic, soldiers exhibited symptom severity levels almost equivalent to the average severity in the pre-pandemic population, regardless of deployment-related quarantining. We did not find gradual differences in the trajectories of psychological distress between the pre- and peri-pandemic samples. Instead, we observed different pre- and peri-pandemic trajectories of psychological distress between two phases of the deployment cycle: two weeks before deployment, which coincided with the start of pre-deployment quarantine, and two weeks post-deployment, which coincided with the end of post-deployment quarantine for the pandemic sample. No difference was found in pre- and peri-pandemic trajectories of psychological distress three to six months post-deployment, compared to two weeks post-deployment. Rather than a delayed onset of symptom severity, psychological distress in deployed soldiers decreased post-deployment. We did not find that deployment-related quarantining increased psychological distress in the pandemic sample compared to the pre-pandemic sample. The main questions warranting explanation are: (1) Why was psychological distress most pronounced pre-deployment for the pandemic sample, in particular already when entering pre-deployment quarantine? (2) Why did symptoms decrease immediately post-deployment for the quarantined soldiers, but did not for soldiers returning home without further confinement? Can these be attributed to post-deployment quarantine?

In regard to the first question, one possible explanation could be that it was not the quarantining experience itself that caused stress symptoms, but rather negative expectations concerning the two weeks of quarantine. This would align with the basic principles of cognitive behavioural therapy (CBT), which suggests that it is not necessarily the objective situation causing stress, but rather the way we think about the situation that contributes to stress [48, 49]. This interpretation is supported by a comparison of the items on the military quarantining adherence scales (MQAS) [50]: No difference was found between expectations regarding difficulties in adhering to the quarantine protocol and the actual experience [23, 24].

Although we did not investigate concerns regarding what a deployment affected by pandemic-related restrictions might entail, these expectations may have played a role. Additionally, the immediate weeks prior to the in-processing into pre-deployment quarantine, could have been stressful, as, in addition to preparing for deployment, many work-related duties and personal tasks at home needed to be completed before leaving for an extended period.

As to the second question, why symptoms decreased immediately post-deployment for the quarantined soldiers, but did not for soldiers returning home without further confinement: At-home quarantining might have been framed in a positive way in line with research that pointed out self-reported positive aspects of the pandemic such as better work-life balance and reduced daily hassles [51–53]. The result might be counter-intuitive when interpreted in the framework of quarantining studies as an infringement on one’s freedom, associated with a potential financial threat due to a loss of income, etc. However, home-based quarantining after months away under restricted living conditions of deployment, might have been welcomed as decompression before starting to work again, as additional time-off which is paid for or in the words of one soldier „being granted an extra holiday “.

However, early post-deployment recovery from stress reactions for the peri-pandemic group as compared to a delayed recovery for the pre-pandemic group may have been linked to different deployment scenarios and a lower number of related traumatic incidents for the pandemic sample. This is suggested by the descriptive comparison of deployment-related traumatic events (see Table 4). Nevertheless, some caution is warranted due to concerns over the reliability and validity of the reported traumatic events. Consequently, ‘exposure to deployment-related traumatic events’ was excluded as a covariate in the main analysis. In a more recent study, the average test–retest reliability of the LEC-5 (κs = 0.4–0.6) was deemed insufficient for reliably distinguishing between trauma-exposed and non-exposed individuals [46], contrasting with previous results of the higher reliability of the LEC-4 [47], which we had relied on when designing the study. A more objective approach would involve using registered events and personnel data from the German Operations Command (see Table 3), although this method has limitations in attributing traumatic events to individual study participants. Furthermore, it does not allow to draw clear-cut conclusions as to which sample has been affected more by deployment-related traumatic events: Comparisons between the pre- and peri-pandemic periods revealed a higher number of events in the pre-pandemic sample (ISAF/RS: 2013–2016), while a greater number of German military personnel were involved in events during the peri-pandemic period (MINUSMA: 2021–2022).

The finding that the psychological distress of deploying soldiers was higher during the pandemic than before aligns with much of the initial research conducted during the pandemic, which found increased depressive and anxiety symptomatology at the onset, often based on cross-sectional studies; one meta-analysis found a significant but small increase in mental health symptomatology [54]. It is contrary to more recent evidence of adaptation to the pandemic and recovery from stress reactions from the first to the second wave [52], though not to pre-pandemic levels [52, 54]. Representative, longitudinal data including pre-pandemic data tend to show smaller negative effects of the pandemic than initial cross-sectional studies with smaller convenience samples [20, 55, 56]. As we did not measure pandemic-related stressors outside quarantine, our explanations for the higher peri-pandemic stress levels are merely hypothetic: Accumulated pandemic stressors, in particular during the first and second wave of the pandemic, concern for family and operational unpredictability could have influenced higher peri-pandemic distress levels.

### Limitations and strengths

The strengths of this study lie in its prospective design, which analysed the impact of accumulated quarantining controlling for the impact of the pandemic and deployment phases: It incorporated not only pre-pandemic reference data but also a reference trajectory of psychological distress over a comparable timeline—specifically, the deployment cycle—while controlling for deployment-related variables, including accumulated deployment experience, length of deployment, and sociodemographic factors such as age, sex/gender, rank (as a proxy for socio-economic status and education). In addition, a number of quarantine-related stress factors were controlled for, in particular the traumatic stress factor of a life-threatening infection and financial harm. Additionally, gender-specific norms for the BSI and Mini-SCL were utilised, enabling the data to be normed accordingly.

Main limitations include the inability to control for deployment-related traumatic events in the main analysis and to control for deployment area and missions, e.g., primarily ISAF/RS in Afghanistan for the pre-pandemic sample and Mali/MINUSMA for the pandemic sample. Although our study controlled for the pandemic period and deployment phases, it has not been possible to completely disentangle the effect of quarantining from the pandemic: This would have required the establishment of a control group without quarantining during the pandemic. We did not measure specific pandemic- or deployment-related stressors outside of quarantine, e.g. accumulated pandemic stressors during the first and second wave of the pandemic, concern for family or operational unpredictability, which limits the explanation of the higher peri-pandemic stress levels.

Although the proportion of female soldiers in our samples was representative of the population of deployed female soldiers, approximately 8–10%, the absolute number in our study did not provide sufficient power for testing the hypotheses for this subgroup. Though high attrition rates, far exceeding 10%, are not unusual for longitudinal studies with military samples and the Little’s MCAR test indicating that missing values were completely at random, there remains a risk of bias in long-term follow-up data due to differential retention.

Another limitation concerns the interpretation of effect sizes for the difference in psychological distress between the pre-pandemic and peri-pandemic periods. The sample size is small, which limits its representativeness. Norms for both the BSI and Mini-SCL were derived from the general population, and the lack of military-specific norms may obscure key risk and resilience factors unique to this group. Additionally, the Mini-SCL, a shorter version of the BSI, uses a different adult population for norming. For the BSI, no stratification by age groups was available, and as a result, only gender-specific T-values were used for both samples.

### Future directions

Future research on mental health and psychosocial well-being across the deployment cycle should focus on two key areas: 1) the development of objective and reliable measures of traumatic events, including combat-related incidents, using brief scales; and 2) the use of larger samples in surveys, with short, streamlined questionnaires that include gender- and age-specific norms, allowing for T-transformation of the data. Ideally, military-specific norms should be established, considering the unique risk and resilience factors in this population.

Although interest in studying the impact of the pandemic and pandemic-related measures is waning, it is important to draw lessons from this experience. These lessons can inform how research in non-pandemic periods should be conceptualised, ensuring that, in the event of future pandemics or other major macro-level stressors, research findings are more valid, representative, and robust. This is particularly important when the effects of single interventions, as in this case quarantining, cannot be isolated using randomised controlled trial designs due to the unique characteristics of such macro-level stressors as the pandemic.

## Data Availability

The datasets generated during the current study are not publicly available as ownership belongs to the German Ministry of Defence. Data are available from the authors on reasonable request (may require data use agreements to be developed).
